# The impact of nocturnal hemodialysis on sexual function

**DOI:** 10.1186/1471-2369-13-67

**Published:** 2012-07-26

**Authors:** Adam Bass, Sofia B Ahmed, Scott Klarenbach, Bruce Culleton, Brenda R Hemmelgarn, Braden Manns

**Affiliations:** 1 Department of Medicine, University of Calgary, Calgary, Alberta, Canada; 2 Libin Cardiovascular Institute, Calgary, Alberta, Canada; 3 Department of Medicine, University of Alberta, Edmonton, Alberta, Canada; 4 Department of Community Health Sciences, University of Calgary, Calgary, Alberta, Canada; 5 Foothills Medical Center, 1403-29th Street NW, Calgary, Alberta, T2N 2 T9, Canada

**Keywords:** Nocturnal hemodialysis, Sex, Sexual function, Frequent hemodialysis

## Abstract

**Background:**

Sexual dysfunction is common in patients with end stage renal disease (ESRD) and treatment options are limited. Observational studies suggest that nocturnal hemodialysis may improve sexual function. We compared sexual activity and responses to sexual related questions in the Kidney Disease Quality of Life Short Form questionnaire among patients randomized to frequent nocturnal or thrice weekly conventional hemodialysis.

**Methods:**

We performed a secondary analysis of data from an RCT which enrolled 51 patients comparing frequent nocturnal and conventional thrice weekly hemodialysis. Sexual activity and responses to sexual related questions were assessed at baseline and six months using relevant questions from the Kidney Disease Quality of Life Short Form questionnaire.

**Results:**

Overall, there was no difference in sexual activity, or the extent to which people were bothered by the impact of kidney disease on their sex life between the two groups between randomization and 6 months. However, women and patients age < 60 who were randomized to frequent nocturnal hemodialysis were less bothered by the impact of kidney disease on their sex life at 6 months, compared with patients allocated to conventional hemodialysis (p = 0.005 and p = 0.024 respectively).

**Conclusions:**

Our results suggest that frequent nocturnal hemodialysis is not associated with an improvement in sexual activity in all patients but might have an effect on the burden of kidney disease on sex life in women and patients less than 60 years of age. The validity of these subgroup findings require confirmation in future RCTs.

## Background

Sexual dysfunction is common in men and women with end stage renal disease (ESRD) [1-5], contributed to by both the diseases that cause ESRD, as well as the consequences of kidney failure [4,6]. Recently, Vecchio et al. published a systematic review examining the treatments available to ESRD patients with sexual dysfunction [7]. Their report highlights the limited treatment options available, noting that phosphodiesterase-5 inhibitors improve erectile dysfunction in men with ESRD, with little research available to guide therapy in women with ESRD.

Frequent nocturnal hemodialysis (NHD) has gained popularity recently as a form of renal replacement therapy, and among other reported benefits, some studies have reported improved sexual function. Published literature however shows conflicting results, although these are based mainly on small observational studies comparing pre-NHD to post-NHD quality of life scores, usually compared with patients on conventional hemodialysis (CvHD) [8-12]. Ting et al. followed 42 patients and noted that sexual function improved after conversion to NHD [11] while Lockridge et al. observed an increase in sexual desire after NHD initiation in 40 patients [10]. However, other studies have not documented improvement, including a recent prospective observational study of 63 patients which demonstrated no improvement in sexual function scores after conversion to NHD [9].

Our group has previously reported a randomized controlled trial in which we examined the effects of NHD on both left ventricular mass as well as quality of life [13-15]. One of the quality of life tools used was the kidney disease quality of life: short form (KDQOL-SF) questionnaire [16], containing specific questions assessing sexual arousal and sexual enjoyment. These questions have not been analyzed nor reported previously. Herein we report the results of a post hoc analysis to determine if frequent NHD was associated with an improvement in sexual activity and responses to sexual related questions listed in the Kidney Disease Quality of Life Short Form questionnaire compared to thrice weekly conventional hemodialysis.

## Methods

### Patients

The methods of this study have previously been reported in detail [15]. Patients were recruited from 10 hemodialysis centers in Alberta, Canada. Patients were considered eligible if they were 18 years old and they were receiving in-center, self-care or home CvHD 3 times a week. In addition to being interested in NHD, patients had to be willing to train and start NHD. Exclusion criteria included physical or mental impediment to training for NHD. Ethics approval was obtained from the Conjoint Health Research Ethics Board at the University of Calgary and informed consent was obtained from all participants.

Fifty-one patients were randomized to either frequent NHD or CvHD in a two group parallel design. Patients randomized to frequent NHD were trained to perform NHD at home for 5-6 nights per week at a minimum of 6 hours, while those randomized to CvHD continued thrice weekly conventional hemodialysis [14]. For the majority of patients treated with CvHD, dialysis was delivered in-centre. Quality of life questionnaires were administered prior to randomization, at the baseline study visit (corresponding to the first day of NHD training for the NHD group), and at study end at 6 months.

### Outcome measures

There are many relevant measures of sexual function, which are outlined below in Table 1. This table also includes a list of the measures that we were able to assess and compares them to contemporary measure of sexual function. The primary outcome that we used was whether patients were sexually active or not, which is easily assessable, though does not capture all relevant domains of sexual function. The secondary outcomes (sexual function, enjoyment and burden of kidney disease on sex life) were measured with questions included within the KDQOL-SF [16]. The questions have been previously validated [17] as a reasonable proxy of sexual function. The relevant questions from the KDQOL-SF were as follows:

**Table 1:** Description of Contemporary Measures of Quality of Sexual Function in comparison to the KDQOL-SF

***Inventory Name***	***Modality/gender***	***Number of items***	***Domains***
Kidney Disease Quality of Life Short Form (KDQOL-SF) ***Present Study	SR/male and female	4	Sexual activity, satisfaction, arousal, burden of kidney disease on sex life
**Arizona sexual experience scale (ASEX)**	SR/male and female	5	Drive, arousal, penile erection/vaginal lubrication, orgasm, satisfaction
**Center for Marital and Sexual Health Sexual Functioning questionnaire (CMASH-SFQ)**	SR/male and partner	21	Sexual frequency, sexual satisfaction, orgasm, erectile function
**Derogatis interview for sexual functioning (DISF-SR)**	CI and SR/male and female	25	Cognition, arousal, behaviour, orgasm, drive/relationship, overall total score
**Female sexual function index (FSFI)**	SR/female only	19	Desire, arousal, lubrication, orgasm, satisfaction, pain
**Index of premature ejaculation**	SR/male only	10	Sexual satisfaction, control, distress
**International index of erectile function (IIEF)**	SR/male only	15	Erectile function, orgasm, desire, intercourse satisfaction, overall total score
**Profile of female sexual function (PFSF)**	SR/female only	37	Desire, arousal, orgasm, pleasure, concerns, responsiveness, self-image
**Sexual function questionnaire (SFQ)**	SR/female only	26	Desire, arousal–sensation, arousal–lubrication, enjoyment, orgasm, dyspareunia, partner relationship, overall total score
**Sexual interest and desire inventory**	CI	13	Overall total score
**Short scale to measure female sexual functioning (SPEQ)**	SR/female only	9	Feelings for partner, sexual responsivity, sexual frequency, libido, dyspareunia, partner problems
**Female sexual distress scale (FSDS/FSDS-R)**	SR/female only	12/13	Unidimensional scale measuring sexually related personal distress. 'R' version has an additional desire item

Abbreviations: CI = clinical interview; SR = self report (Adapted with permission from Derogatis [26]).

Sexual activity and enjoyment questions:

1. Have you had any sexual activity in the past 4 weeks? (yes / no)

2. If yes, then how much of a problem was each of the following in the past 4 weeks?Burden of kidney disease on sex life question (answered by all participants):

A.) Enjoying sex?

B.) Becoming sexually aroused?

3. Some people are bothered by the effects of kidney disease on their daily life, while others are not. How much does kidney disease bother you … with respect to…Your sex life?

The responses to the two sexual enjoyment and arousal questions and the burden of kidney disease on sex life question were recorded on a five point Likert scale, ranging from 1 to 5 (1 representing no problem or not bothered and 5 indicating a severe problem or extremely bothered). For the secondary outcomes, we chose to examine the proportion of patients who reported improvement. The burden of kidney disease on sex life question was answered and analysed in all patients, while the sexual enjoyment and arousal questions were answered and analysed only in patients who had sexual activity in the prior 4 weeks.

### Statistical analysis

To confirm the reliability of the sexual arousal and enjoyment question, Cronbach’s alpha was calculated on these questions in patients who declared that they had engaged in sexual activity. In addition, to verify the validity of analyzing the burden of kidney disease on sex life question alone, Pearson’s R correlation coefficient was determined between this question and the sexual enjoyment and arousal question in those participants who had engaged in sexual activity. A Cronbach’s alpha was also calculated between the three questions together to determine if taken together, they demonstrate reliability in measuring sexually related concerns.

All analyses used the intention to treat principle and all enrolled patients were included in the analysis. For the primary outcome (sexual activity), we first assessed whether there was a higher proportion of patients reporting sexual activity using Chi square tests. We next compared changes in burden of kidney disease scores from randomization to study end (6 months) between patients allocated to frequent NHD and conventional hemodialysis. Since there is no requirement in the KDOQL-SF for a patient to be engaged in sexual activity to answer the burden of kidney disease on sex life question, and since we were interested in whether NHD reduced the burden of kidney disease on sex life – an outcome that is relevant, irrespective of sexual activity, we chose to analyze all patients irrespective of sexual activity. Given that we were testing differences in proportions, we used chi-square tests and applied the Yates continuity correction for analyses that did not meet the 5 items expected per cell criteria. For missing data at six months (n = 3; due to death or loss to transplantation), we used the last value carried forward approach [14].

Among the subgroup of patients reporting sexual activity, we next categorized patients based on whether they experienced an improvement for both the domains “enjoying sex”, or “becoming sexually aroused”. This was chosen because it is considered a clinically significant change [13]. Statistical significance was defined as a p-value < 0.05.

Since the causes and prevalence of sexual dysfunction differs across patients with ESRD, we performed exploratory analyses to determine if there was any subpopulation of patients who might benefit from nocturnal hemodialysis with respect to sexual function. All subgroups were conceived prior to analyzing the data. These included women, patients <60 years of age, patients without vascular disease, and patients without diabetes. It was not possible to analyze any subgroups for the sexual activity question due to the small numbers of patients who were sexually active. However, we chose to do subgroup analysis on the burden of kidney disease question since there was no requirement to be sexually active to answer the question. All statistical analyses were performed using STATA software package version 11.

## Results

The baseline characteristics between the two groups are listed in Table 2. Fifty-one patients answered the QOL questionnaire at randomization and 48 at study end. No significant differences in baseline characteristics were detected between the two groups. Approximately 1/3 of patients had diabetes and the cohort was relatively young with an average age of 55 in the NHD group and 53 in the CvHD group. The majority of patients who participated in the study were Caucasian and a greater proportion of participants were male than female. There was no difference in the number of patients in relationships in either group with 72% with a partner in the NHD group and 73% in the CvHD group.

**Table 2:** Baseline characteristics at study initiation by dialysis modality

**Characteristic**	**Nocturnal hemodialysis (n = 26)**	**Conventional hemodialysis (n = 25)**		
*Age (years)*	55.1 ± 12.4	53.1 ± 13.4		
*Male gender (%)*	18 (69)	14 (56)		
*Caucasian (%)*	23 (88)	21 (84)		
*Time on dialysis (years)*	5.5 ± 5.3	4.8 ± 3.8		
*Median (interquartile range)*	3 (1–9)	4 (2–6)		
**Baseline dialysis modality (%)**				
*In-center hemodialysis*	18 (69)	13 (52)		
*Home or self-care hemodialysis*	2 (8)	5 (20)		
*Home hemodialysis*	6 (23)	7 (28)		
**Cause of ESRD (%)**				
*Diabetic nephropathy*	7 (27)	8 (32)		
*Hypertension/vascular*	2 (8)	2 (8)		
*Glomerulonephritis*	5 (19)	8 (32)		
*Polycystic kidney disease*	3 (12)	1 (4)		
*Urologic*	3 (12)	3 (12)		
*Other*	6 (24)	3 (12)		
**Comorbid illnesses (%)**				
*Ischemic heart disease*	10 (38)	10 (40)		
*Congestive heart failure*	6 (23)	5 (20)		
*Peripheral vascular disease*	4 (15)	4 (16)		
*Cerebrovascular disease*	5 (19)	3 (12)		
*Diabetes mellitus*	10 (38)	11 (44)		
**β-Blocker usage (%)**	10 (38)	9 (36)		
**Married, Common-law or in**	18 (72)	19 (73)		
**Relationship (%)**				
**Responses to relevant sexual questions**	Baseline	**6 Months**	Baseline	**6 Months**
*Proportion with Sexual Activity in Last 4 Weeks*	36%	32%	38%	31%
*Proportion of patients very much or extremely bothered on burden of Kidney Disease on Sex Life*	60%	39%	50%	40%
Proportion of patients having sex who reported very much or severe problems enjoying Sex*	55%	70%	40%	88%
Proportion of patients having sex who reported very much or severe problem becoming Sexually Aroused*	55%	70%	40%	88%

ESRD, end-stage renal disease.

Values (±) are means ± standard deviation.

P > 0.05 for all comparisons between nocturnal hemodialysis and conventional.

*proportion of patients engaging in sexual activity.

The Cronbach’s alpha of the sexual arousal and enjoyment questions was 0.97 in patients who had engaged in sexual activity indicating excellent reliability of these questions. The Pearson R correlation coefficient of the burden of kidney disease on sex life question was 0.61 (p = 0.01) with both the sexual enjoyment and arousal questions indicating a strong correlation between these questions. The Cronbach’s alpha of all three questions analyzed together was 0.89 suggesting good reliability.

**Figure 1:**
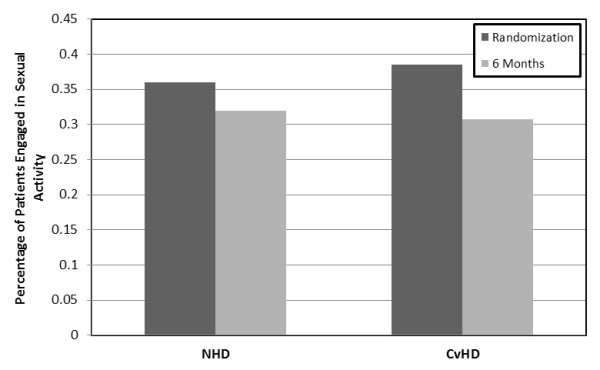
Percentage of patients reporting sexual activity by dialysis modality at randomization and at six months.

With respect to our primary outcome, there was no change in the proportion of patients who reported being sexually active at six months compared to randomization (Figure 1). Of the patients with recent sexual activity, there was also no significant difference between the two groups at 6 months when changes in the proportion of patients enjoying sex or becoming sexually aroused were considered.

**Figure 2:**
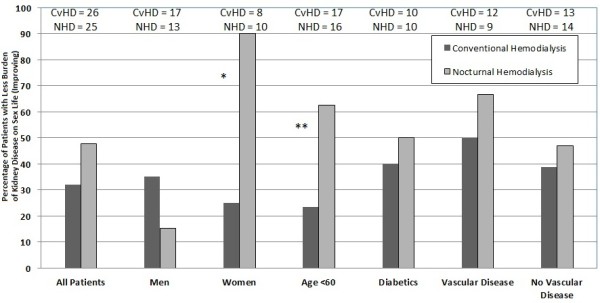
**Proportion of patients showing improvement in burden of kidney disease on sex life.** Listed above the columns are the number of patients in each group according to modality. All categories not statistically significantly different except Women (*p = 0.02) and Age < 60 (**p = 0.02).

For the burden of kidney disease on sex life question (a measure of sexual function), at six months, 39% patients in the NHD group had scores indicating that they were not at all bothered to moderately bothered by sexual dysfunction, compared with 60% in the CvHD group (*p* = 0.28). When comparing the burden of kidney disease scores, 45% and 32% of NHD and conventional hemodialysis patients, respectively, experienced an improvement by one category in their scores (*p* = 0.2) (Figure 2). In women and patients below the age of 60 years old, there was a statistically significant improvement in burden of kidney disease on sex life scores for patients allocated to nocturnal hemodialysis (*χ*^2^ = 7.90, *p* = 0.02 and *χ*^2^ = 5.12 *p* = 0.02) (Figure. 2).

## Discussion

In this post-hoc analysis of a randomized controlled trial we observed no improvement in sexual activity or or self-reported arousal, enjoyment or burden of kidney disease on sex life between patients on NHD versus CvHD overall. Consistent with prior reports of sexual activity on hemodialysis, only 43 percent of patients indicated that they were sexually active in the 4 weeks prior to either randomization or study completion [3]. Our findings are not consistent with prior research suggesting that NHD may improve sexual activity and sexually related concerns.

Normal sexual function involves a complex interplay between the hormonal, vascular, neurological and psychological systems. These can all be impacted by ESRD. Patients with ESRD have abnormalities in the hypothalamic-pituitary axis, in particular, hypogonadism and hyperprolactinemia which are thought to be secondary to the accumulation of uremic toxins [18]. The diseases that cause ESRD and the condition itself can cause vascular insufficiency as well as sensory and autonomic neuropathy [4]. Finally, the complex social and psychological factors that are embroiled with ESRD impact normal sexual function [19]. Given this, the mechanism by which NHD might improve sexual function in ESRD patients is not clear. Since NHD improves clearance of uremic toxins it might improve hormonal dysfunction, but it may not address other inhibitors of normal sexual function. NHD does not alleviate the comorbid conditions that cause ESRD which are well known to cause sexual dysfunction. Specifically, NHD is unlikely to reverse pre-existing vascular and neurological damage causing impairment. In addition, it may have variable effects on the social and psychological dynamics caused by ESRD. In particular, sexual activity might be either positively or negatively affected by undergoing dialysis in the home. Transplantation provides a good example of the potential multi-factorial nature of sexual dysfunction in that despite normalization of kidney function, many patients still experience sexual dysfunction [20].

While we did not note any improvement with NHD on sexual activity or self-reported arousal, enjoyment or burden of kidney disease on sex life overall, a subgroup analysis suggested that patients less than 60 years of age and women were less burdened by the effect of their kidney disease on their sex life after 6 months of NHD. It is plausible that younger patients might be more likely to experience benefit with NHD since it is conceivable that they might have fewer and less severe comorbid diseases, which themselves may impair sexual function. It is uncertain why women might benefit from NHD with respect to the burden of kidney disease of sex life. While both sexes suffer from decreased libido, there are differences in sexual dysfunction between men and women with ESRD with men generally suffering from impotence [4] and women suffering from anorgasmia, decreased lubrication and dyspareunia [21]. NHD may have no impact on impotence in men, while it is possible that women perceive NHD to be less intrusive on normal sexual function than CvHD. While this study did not specifically measure these outcomes, it is possible that changes in these domains could have impacted the burden of kidney disease on sex-life in these sub-groups. Alternatively, while the burden kidney disease on sex life diminished in these patients, there are other burdens aside from kidney disease that may be impacting proper sexual activity and function which may explain why we did not observe a concomitant increase in sexual activity.

Our study is the first randomized controlled trial documenting the association between NHD and sexual activity and self-reported sexual function. However, it has several limitations which should be considered. The original RCT was not specifically designed to examine the impact of NHD on sexual function. While the KDQOL-SF does include domains designed to measure sexual function and activity, it would have been preferable to use dedicated sexual function scales such as the International Index of Erectile Function [22] or the Female Sexual Function Index [23]. Despite this, we feel that our study provides much needed insight into a poorly studied area that is pervasive in dialysis patients. Other limitations include that the RCT was not powered to detect differences in quality of life, and that all analyses documented herein are posthoc and exploratory in nature. Given the limited number of patients and that few patients answered the sexual function questions, our subgroup findings should be interpreted with particular caution. However, our findings can be tested within secondary analyses of other recently reported randomized trials of frequent hemodialysis [24,25]. It should also be noted that given the nature of the intervention it is highly unlikely that a randomized controlled trial focussed on this particular outcome will ever be undertaken.

## Conclusions

In conclusion, our study is the first randomized controlled trial examining the effect of NHD on sexual activity or self-reported sexual arousal, enjoyment or burden of kidney disease on sex life. While NHD does not appear to improve sexual activity overall, women and patients younger than 60 years old might experience improvement - the validity of the subgroup findings should be assessed in future RCTs.

## Competing interests

A.B., S.B.A., S. K., B.R.H.and B.J.M. declare that they have no competing interests. B.F.C. had no conflicts of interest to declare at the time this research was conducted. He is now employed by and has shares in Baxter Healthcare. Baxter Healthcare provided no funding and had no input into the analysis or interpretation of the results and no input into the drafting of the manuscript.

## Authors’ contributions

A.B. contributed to data analysis, interpretation of the data, and drafted the manuscript S.B.A. contributed to interpretation of the data and drafting of the manuscript, S. K. contributed to study conception and design, interpretation of the data and drafting of the manuscript, B.R.H. contributed to study conception and design, interpretation of the data and drafting of the manuscript, B.J.M. contributed to study conception and design, data analysis, interpretation of the data, and drafted the manuscript B.F.C. contributed to study conception and design, interpretation of the data and drafting of the manuscript. All authors read and approved the final manuscript.

## Pre-publication history

The pre-publication history for this paper can be accessed here:

http://www.biomedcentral.com/1471-2369/13/67/prepub
